# Single Nucleotide Polymorphisms in *P2X7* Gene Are Associated with Serum Immunoglobulin G Responses to *Mycobacterium tuberculosis* in Tuberculosis Patients

**DOI:** 10.1155/2015/671272

**Published:** 2015-12-20

**Authors:** Jiangdong Wu, Lijun Lu, Le Zhang, Yulei Ding, Fang Wu, Weize Zuo, Wanjiang Zhang

**Affiliations:** Key Laboratory of Xinjiang Endemic and Ethnic Diseases, Cooperated by Education Ministry with Xinjiang Province, Shihezi University, Shihezi 832000, China

## Abstract

*Objective*. Our study investigated the association between single nucleotide polymorphisms (SNPs) in P2X7 gene and serum immunoglobulin G (IgG) responses to mycobacterium tuberculosis (MTB) in TB patients. *Methods*. A total of 103 TB patients were enrolled as case group and 87 healthy individuals at same geographical region as control group. The SNP detection of 1513A>C and -762T>C was performed using PCR-RFLP, and the levels of serum IgG responses to MTB in all subjects were determined. *Results*. AC and CC of 1513A>C and TC and CC of -762T>C had higher frequencies in case group than in control group. TB patients carrying TC and CC of -762T>C had higher positive rate of IgG responses to MTB than those carrying TT. Additionally, patients carrying TC and CC of -762T>C had more MTB in sputum than those carrying TT. *Conclusion*. P2X7 SNPs, 1513A>C and -762T>C, may be associated with the susceptibility to tuberculosis, and -762T>C SNP may contribute to the development of MTB. The mutant genotype of -762T>C (TC and CC) may lower human capability of phagocytosis to MTB, leading to an increased morbidity of TB.

## 1. Introduction

Tuberculosis (TB) is a global public health priority and remains the second leading cause of infection-related mortality worldwide [1]. The World Health Organization (WHO) has estimated that 9 million people were diagnosed with TB and 1.5 million people died of this disease in 2013 (deaths up from 1.3 million estimated in 2012) [2]. TB is largely caused by* Mycobacterium tuberculosis* (MTB) which has infected around a third of the world population, but only 3~10% of those infected progress to active disease in their lifetime, and up to 90% of infected people are asymptomatic with a latent infection [3]. Multiple factors contribute to the risk of infection and development of TB, including environmental factors such as socioeconomic conditions, acute infection and smoking, and genetic factors as well as host-pathogen interactions [4, 5]. Epidemiological studies reported that the development and progression of TB in human is strongly associated with gene polymorphism; up to now, gene polymorphisms of* vitamin D receptor*,* SLC11A1*,* tumor necrosis factor-alpha*,* Toll-like receptor 2*, and* monocyte chemoattractant protein-1* have been identified to be associated with the susceptibility to TB [6–9].

The P2X7 receptor is predominately expressed on hematopoietic, mesenchymal, and epithelial cells and neural lineages, playing a crucial role in immunity, inflammation, neurological function, bone homeostasis, and neoplasia [10]. Human* P2X7* gene containing 13 exons encodes the P2X7 receptor and is located on chromosome position 12q24 which is a region relevant to inflammatory and psychiatric disorders [11]. P2X7 activation induces an array of downstream signaling events, in a cell specific manner, including the release of cell proliferation or death, proinflammatory mediators, and killing of intracellular pathogens [12]. Some studies reported that several single nucleotide polymorphisms (SNPs) in* P2X7* gene result in the reduction or loss of receptor function, and the most common SNPs involve the 1513A>C and -762T>C [13, 14]. Accumulating studies suggested that these two SNPs of* P2X7* gene play an important role in TB susceptibility, while there was no significant association with -762T>C polymorphism [15, 16]. However, some previous studies demonstrated no correlation of* P2X7* gene SNPs with susceptibility to TB [17, 18]. Although there were increasing studies investigating the correlation between* P2X7* gene SNPs and TB, it is rare to explore whether* P2X7* gene SNPs influence immunoglobulin G (IgG) responses to MTB. The detection of antibodies to MTB in a patient's serum is simple and applicable in various settings and provides extremely rapid results [19]. Several studies in humans as well as animal models have reported that anti-MTB titers rely on the state of infection and that they are associated with the degree of mycobacterial burden [20, 21]. It has been demonstrated that antibody to *α*-crystallin is a promising target for the serological diagnosis of patients with active TB patients [22]. Therefore, here we performed a case-control study to determine the association between SNPs in* P2X7* gene and susceptibility to TB and further examine the role of* P2X7* gene SNPs in levels of serum IgG responses to MTB in TB patients.

## 2. Materials and Methods

### 2.1. Ethics Statement

The study was designed in strict consistency with the protocols established by the ethics committee of Key Laboratory of Xinjiang Endemic and Ethnic Diseases Cooperated by Education Ministry with Xinjiang Province, and the informed consents were signed by all participants. All the experimental procedures in this study were performed on the basis of Declaration of Helsinki [23].

### 2.2. Subjects

A total of 103 TB patients (64 males and 39 females; 42 cases of Han, 30 cases of Uygur, and 31 cases of Kazak) with a mean age of 48.6 ± 14.9 years, hospitalized between January 2014 and November 2014 at the Key Laboratory of Xinjiang Endemic and Ethnic Diseases Cooperated by Education Ministry with Xinjiang Province, were enrolled into our study as case group. All the patients were diagnosed as TB according to diagnostic criteria published by Ministry of Health of China (WS288-2008, http://www.moh.gov.cn/zwgkzt/s9491/200801/38801.shtml) or* Draft Global Strategy and Targets for Tuberculosis Prevention*,* Care and Control after 2015* by WHO (http://apps.who.int/gb/ebwha/pdf_files/WHA67/A67_11-en.pdf). The inclusion criteria were as follows: (1) the diagnosis of TB was based on clinical manifestations, bacteria culture, and imaging examination, according to the diagnostic criteria published by Ministry of Health of China; (2) all patients were in primary pulmonary TB. Exclusion criteria were as follows: (1) individuals with similar symptoms of TB; (2) TB patients with complications of chronic obstructive pulmonary diseases (COPD), asthma, pneumonia, cancers, diabetes mellitus or hypertension, and so forth; (3) patients with heredofamilial history; (4) immunocompromised patients (with HIV infection, lipoma or long-term persistence of hormone or organ transplant, etc.). Additionally, our study also enrolled 87 healthy controls (51 male and 36 female; 35 cases of Han, 25 cases of Uygur, and 27 cases of Kazak) with a mean age of 47.0 ± 14.5 years at the same geographic region. There was no statistical difference in age, gender, and ethnic constitution between the case and control groups (all* P* > 0.05).

### 2.3. Sample Collection

Peripheral blood (10 mL) was collected from all subjects in the morning after fasting for 10 to 12 h and placed into two tubes (5 mL/tube). The first 5 mL was added with ethylenediaminetetraacetic acid (EDTA) for anticoagulation. After naturally cooling to room temperature for one hour later, the mixture was centrifuged at 3000 rpm for 10 min to separate peripheral blood mononuclear cell. DNA was extracted from peripheral white blood cells using a DNA extraction kit (Beijing Tiangen Biotechnology Co., Ltd., China, DP318-03). Then the other 5 mL peripheral blood was centrifuged at 3000 rpm for 10 min at 4°C to collect supernatant. Subsequently, the supernatant was centrifuged again at 12,000 rpm for 5 min at 4°C to extract serum. The serum was subpackaged into 200 *μ*L tubes and then stored at −80°C in refrigerator for further usage.

### 2.4. SNP Detection

Two SNPs (1513A>C and -762T>C) in* P2X7* gene were selected for research targets. Polymerase chain reaction-restriction fragment length polymorphism (PCR-RFLP) was used for analyzing 1513A>C and PCR for -762T>C SNPs. PCR primers were designed by Premier 5.0 software (http://www.premierbiosoft.com/) and synthetized by Shanghai biological engineering company. The SNP-amplified, primer sequences, fragment length, annealing temperature, and cycle number were listed in Table 1. PCR was set up in a volume of 20 *μ*L, containing 2 *μ*L 10x PCR buffer, 2 *μ*L deoxyribonucleoside triphosphate (dNTP) (each for 2.5 mmol/L), 0.5 *μ*L (10 pmol/*μ*L) each upstream and downstream primer, 2.5 U Platinum Taq DNA polymerase (Invitrogen Biotechnology Co., Ltd., Shanghai, China), and 50 ng genome DNA. The PCR amplification for 1513A>C included initial denaturation at 95°C for 3 min, denaturation at 94°C for 50 s, annealing at 64°C for 45 s, and extension at 72°C for 30 s, with a total of 35 cycles, followed by the final extension at 72°C for 10 min. The amplified fragment length of 316 bp was obtained. The PCR product was digested at 37°C for 3 h with Hae II (Promega, America) and the product underwent electrophoresis in a 1.5% agarose gel to determine the genotype. The PCR amplification for -762T>C was as follows: initial prenaturation at 95°C for 5 min, denaturation at 94°C for 20 s, annealing at 65°C for 30 s, and extension at 72°C for 30 s, with a total of 10 cycles, followed by the final extension at 72°C for 10 min. The amplification products were visualized by 1.5% agarose gel electrophoresis to determine the genotype.

**Figure 1:**
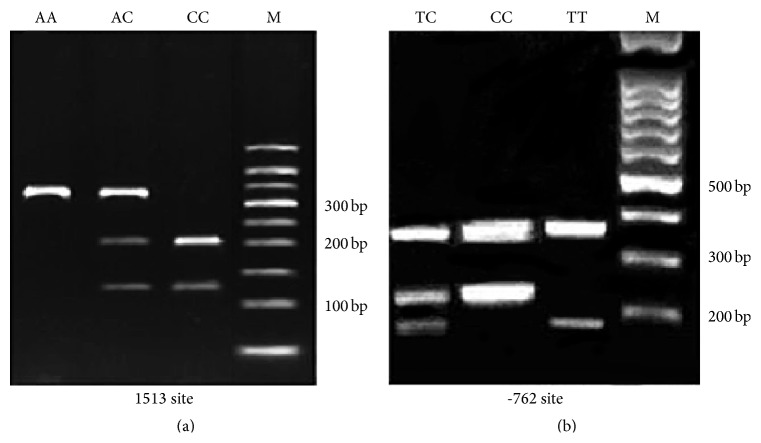
The electropherograms of 1513A>C and 762T>C SNPs in* P2X7* gene after enzyme digestion and polymerase chain reaction (PCR) ((a) 1513 site; (b) -762 site; note: PCR products of the two polymorphic sites were consistent with expectancy, and genotypes were confirmed by gel electrophoresis after digestion with restriction enzyme).

The electropherograms of 1513 and -762 sites in* P2X7* gene after enzyme digestion and PCR were presented in Figure 1. As shown in Figure 1(a), when 1513 site was AA, there was no restriction enzyme site and the fragment length was 316 bp after restriction enzyme digestion by Hae II. When 1513 site was CC, 199 bp and 120 bp fragments were obtained. When 1513 site was AC, 316 bp, 199 bp, and 120 bp fragments were exhibited. As shown in Figure 1(b), when -762 site was CC, the PCR products were 373 bp and 235 bp. When -762 site was TT, the PCR products were 373 bp and 186 bp. When -762 site was TC, the PCR products were 373 bp, 235 bp, and 186 bp.

### 2.5. Detection of Serum IgG Responses to* MTB* Antigens

Protein chip kit for detection of IgG responses to MTB (Nanjing Potomac Bio-Technology Co., Ltd., China, S20050089) was adopted, using three MTB complex specific antigens (16 kDa, 38 kDa, and lipoarabinomannan (LAM)) to determinate IgG responses to MTB in serum. The result was considered positive if one of the three antibodies was detected positive; otherwise the result was considered negative. Sputum from patients was mounted on slide. After acid-fast staining, the slide was examined under light microscope. The number and distribution of cells were observed under low magnification (400x), and even distribution and great morphology were observed again under oil-immersion lens (1000x) for acid-fast bacillus. Continuous observation of no piece (100x) twice was considered as acid-fast bacilli negative and continuous observation of 3~9 pieces (100x) twice as acid-fast bacilli positive.

### 2.6. Statistical Analysis

All data were analyzed by SPSS 18.0 software (IBM Corporation, Somers, NY, USA). Continuous data were expressed as mean ± standard deviation ($\overset{-}{x}\;\;$± SD), and* t*-test or variance analysis was performed to detect intergroup comparisons. Categorical data were expressed as percentage, which were compared by *χ* ^2^ test and Fisher's exact test. Hardy-Weinberg equilibrium test was applied to detect whether the two groups were representative. In addition, SHEsis software was used to analyze whether these two SNPs were in linkage disequilibrium. Genotype frequencies comparisons between groups were presented as odds ratio (OR) and 95% confidence interval (CI). All* P* values were two-sided, and the level of significance was set at* P* < 0.05.

## 3. Results

### 3.1. Distributions of* P2X7* SNPs

Genotype frequency distributions of 1513A>C and -762T>C in two groups were consistent with Hardy-Weinberg equilibrium, which provided evidence of group representativeness for these two SNPs (all* P* > 0.05). These two SNPs were in linkage disequilibrium with 1.000 of *D*′ and 0.726 of *r* ^2^.  Table 2 presented the comparison of allele frequency of 1513A>C and -762T>C SNPs between case and control groups. AC and CC of 1513A>C and TC and CC of -762T>C in case group had higher frequency than control group (all* P* < 0.05). Both AC and CC of 1513A>C were risk factors of TB, and C was risk allele of TB (AC: OR = 2.805, 95% CI = 1.475~5.332; CC: OR = 3.606, 95% CI = 1.473~8.829; C: OR = 2.268, 95% CI = 1.466~3.511). Additionally, both TC and CC of -762T>C were risk factors of TB, and C was risk allele of TB (AC: OR = 3.581, 95% CI = 1.800~7.123; CC: OR = 8.889, 95% CI = 3.634~21.74; C: OR = 3.451, 95% CI = 2.240~5.315).

### 3.2. Serum IgG Responses to* MTB* Antigens

The data in Table 2 suggested that 1513A>C and -762T>C SNPs may correlate to the susceptibility to TB. The positive rates of IgG responses to MTB in different SNPs of case group were identified, which was shown in Table 3. TB patients carrying TC and CC of -762T>C had higher positive rate of IgG responses to MTB than those carrying TT (TC:* P* = 0.008; CC:* P* = 0.030). However, there was no significant difference about the positive rate of IgG responses to MTB antigens between patients carrying AC and CC of 1513A>C and those carrying AA (all* P* > 0.05).

### 3.3. Examination under the Light Microscope

**Figure 2:**
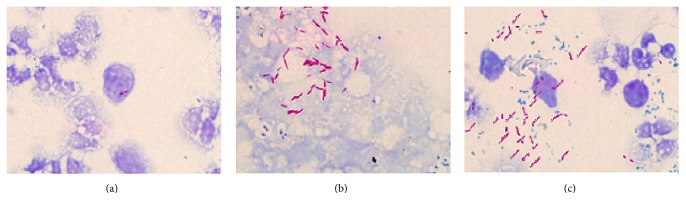
Examination of sputum with* Mycobacterium tuberculosis* (MTB) positive samples in -762T>C single nucleotide polymorphism (SNP) of* P2X7* gene under the light microscope ((a) TT genotype; (b) TC genotype; (c) CC genotype).

As shown in Table 3, -762T>C SNP in* P2X7* gene was associated with the level of serum IgG responses to MTB. Examination under the light microscope was visualized in Figure 2, from which we found that positive rates of acid-fast bacilli were 19.0% in sputum of TT carriers, 48.9% of TC carriers, and 57.1% of CC carriers, and TC (Figure 2(b)) and CC (Figure 2(c)) carriers of -762T>C had greater MTB in sputum than those TT carriers (Table 4).

## 4. Discussion

In the present study, we evaluated whether* P2X7* polymorphism confers susceptibility to TB and investigated the association of SNPs in* P2X7* gene with levels of serum IgG responses to MTB antigen in TB patients. Our results suggested that 1513A>C and -762T>C SNPs may be a risk factor for the development and progression of TB. The mutant genotype of -762T>C (TC and CC) may lower human capability of phagocytosis to MTB, leading to an increased morbidity of TB.

One of our findings showed that an increased frequency of AC and CC of 1513A>C and TC and CC of -762T>C was observed in TB patients, suggesting that SNPs in* P2X7* gene were associated with TB. Development and progression of TB is a result of complicated host-pathogen interactions involving many elements in the innate together with adaptive immune systems [24]. Macrophages are the primary host cells for intracellular replication of mycobacteria and participate in controlling human infection, which serve as antigen presenting cells in reactivation of lymphocytes at the infection sites [25]. Extracellular adenosine triphosphate (ATP) mediates the germicidal activity of macrophages by activation of the P2X7 receptor resulting in apoptosis of the macrophage, which functions as a crucial host defense mechanism against MTB infection [26]. The purinergic P2X7 receptor is an ATP-gated cation channel expressed in immune cells, which affects the release of proinflammatory cytokines from monocytes and macrophages [27]. Collectively, the P2X7 receptor is highly expressed on the macrophage cell surface and activities of infected cells via extracellular ATP have been demonstrated to kill intracellular bacteria and parasites [28].

The* P2X7* gene is highly polymorphic and a variety of nonsynonymous SNPs have been reported which were involved in alternation of receptor function or expression, especially 1513A>C and -762T>C SNPs [13]. A previous study published by Gartland and his colleagues reported 50% reduction in monocyte P2X7 receptor function in 1513C compound heterozygotes as well as complete loss of function for 1513C homozygote [29]. Fernando et al. also suggested that the 1513A>C SNP enhances susceptibility to TB, and this defect is correlated to the reduction in the capacity of macrophages to kill MTB [30]. In addition, -762T>C transformation may downregulate function of the binding site of some transcription factor in sequence, including CCAAT enhancer binding protein and GATA binding factor, inducing the decline of P2X7 receptor function [25]. In our study, patients carrying TC and CC of -762T>C had greater MTB in sputum than those carrying TT. Therefore,* P2X7* gene SNPs cause the reduction of P2X7 receptor function, setting a barrier in the bonding of P2X7 receptor with ATP, thereby reducing the capacity of macrophages to kill MTB, which was associated with the pathogenesis of TB.

Another important finding of our study was that TB patients carrying TC and CC of -762T>C had higher positive rate of IgG responses to MTB than those carrying TT. In our study, we used three MTB-specific antigens (16 kDa, 38 kDa, and LAM) to determinate antibody (anti-MTB) in serum. The 38 kDa antigen is a secretory, MTB antigen can reach a specificity of as high as 90% for TB diagnosis, and the 16 kDa antigen is an immunodominant antigen and contains B-cell epitopes specific for the MTB complex [31]. LAM, as an inherent constituent of the MTB cell wall, can induce the human body to engender anti-LAM antibodies [32]. Millington et al. have described that antigens which elicit strong T-cell immunity are specific for MTB infection and also are crucial for immunodiagnosis [33]. In our study, an elevated positive rate of IgG responses to MTB was detected in -762T>C SNP. Hur et al. also demonstrated that significantly higher IgG responses to 16 kDa and 38 kDa were observed, which was consistent with our study suggesting that -762T>C SNPs were correlated with the development of MTB [34].

In conclusion, our study demonstrated that 1513A>C and -762T>C SNPs in* P2X7* gene may be associated with the susceptibility to TB, and -762T>C SNP may contribute to the development of MTB. The mutant genotype of -762T>C (TC and CC) may lower human capability of phagocytosis to MTB, leading to an increased morbidity of TB. Functional research including large sample size will need to confirm the role of* P2X7* gene SNPs in the development and progression of TB and the growth of MTB.

## Figures and Tables

**Table 1:** The SNP-amplified, primer sequences, fragment length, annealing temperature, and cycles in *P2X7* gene.

SNP	Primer	Fragment length	Annealing temperature	Cycles
1513 A>C	5′-AGACCTGCGATGGACTTCACAG-3′	316	64	35
	5′-GCCAGGTGGCGTAGCACCTG-3′			

-762 T>C	Outer primer			
	5′-GAAACAGGGCCCTGGGTCCTC-3′	373	65	10
	5′-TGGTGGGGGTGGAGGGGC-3′			
	Inner primer			
	5′-GGTGTCCCTCACTGAATAGGTCAAT-3′	235/186	63	30
	5′-GGCAGTCCAACAAAGTTAGGTTTG-3′			

SNP: single nucleotide polymorphism.

**Table 2:** Genotype frequency distributions of 1513A>C and -762T>C in *P2X7* gene.

Genotype	Control group (*n* = 87)	Case group (*n* = 103)	*P*	OR	95% CI
1513A>C					
AA	51 (58.6%)	33 (32.0%)	Ref.		
AC	27 (31.0%)	49 (47.6%)	0.002	2.805	1.475–5.332
CC	9 (10.4 %)	21 (20.4%)	0.004	3.606	1.473–8.829
Dominant model					
AA	51 (58.6%)	33 (32.0%)	Ref.		
AC + CC	36 (41.4%)	70 (67.6%)	<0.001	3.005	1.658–5.446
Recessive model					
AA + AC	78 (89.6%)	82 (79.6%)	Ref.		
CC	9 (10.4%)	21 (20.4%)	0.06	2.22	0.958–5.143
Allele					
A	129 (74.1%)	115 (55.8%)	Ref.		
C	45 (25.9%)	91 (44.2%)	<0.001	2.268	1.466–3.511
-762T>C					
TT	48 (55.2%)	21 (20.4%)	Ref.		
TC	30 (34.5%)	47 (45.6%)	<0.001	3.581	1.800–7.123
CC	9 (10.3%)	35 (34.0%)	<0.001	8.889	3.634–21.74
Dominant model					
TT	48 (55.2%)	21 (20.4%)	Ref.		
CC + TC	39 (44.8%)	82 (79.6%)	<0.001	4.932	2.598–9.365
Recessive model					
TT + TC	78 (89.7%)	68 (66.0%)	Ref.		
CC	9 (10.3%)	35 (34.0%)	<0.001	4.461	2.001–9.944
Allele					
T	126 (72.4%)	89 (43.3%)	Ref.		
C	48 (27.6%)	117 (56.8%)	<0.001	3.451	2.240–5.315

OR: odds ratio; CI: confidence interval; Ref.: reference.

**Table 3:** Positive rate of immunoglobulin G responses to *Mycobacterium tuberculosis* in 1513A>C and -762T>C of *P2X7* gene in case group.

Genotype	IgG responses to MTB		*P*	OR	95% CI
	Negative	Positive			
1513A>C					
AA (*n* = 33)	11 (33.3%)	22 (66.7%)	Ref.		
AC (*n* = 49)	11 (22.4%)	38 (77.6%)	0.2753	1.727	0.644 to 4.636
CC (*n* = 21)	3 (14.3%)	18 (85.7%)	0.1195	3.000	0.725 to 12.42
-762T>C					
TT (*n* = 21)	10 (47.6%)	11 (52.4%)	Ref.		
TC (*n* = 47)	8 (17.0%)	39 (83.0%)	0.008	4.432	1.409 to 13.94
CC (*n* = 35)	7 (20.0%)	28 (80.0%)	0.030	3.636	1.104 to 11.97

IgG: immunoglobulin G; MTB: *Mycobacterium tuberculosis*; OR: odds ratio; CI: confidence interval; Ref.: reference.

**Table 4:** Examination of sputum under the light microscope in 1513A>C and -762T>C of *P2X7* gene in case group.

	TT	TC	CC	*P* ^*∗*^	*P* ^#^
Positive	4 (19.0%)	23 (48.9%)	20 (57.1%)	0.020	0.005
Negative	17 (81.0%)	24 (51.1%)	15 (42.9%)		

*P* ^*∗*^: TC genotype was compared with TT genotype; *P* ^#^: CC genotype was compared with TT genotype.
